# Studies of lectin binding to normal and neoplastic lymph nodes. II. Non-Hodgkin's lymphoma.

**DOI:** 10.1038/bjc.1982.242

**Published:** 1982-10

**Authors:** V. H. Bramwell, D. Crowther, J. Gallagher, R. W. Stoddart

## Abstract

**Images:**


Br. J. Cancer (1982) 46, 582

### STUDIES OF LECTIN BINDING TO NORMAL AND NEOPLASTIC LYMPH NODES. II. NON-HODGKIN'S LYMPHOMA

V. H. C. BRAMWELL*, D. CROWTHER*, J. GALLAGHER* AND R. W. STODDART†

*From the *CRC Department of Medical Oncology, University of Manchester and Christie Hospital and Holt Radium Institute, Wilmslow Road, Manchester M20 9BX, and the †Department of Experimental Pathology, Stopford Building, University of Manchester, Manchester 13*

Received 11 January 1981 Accepted 25 June 1982

**Summary.—Fluorescein conjugated lectins have been used as histochemical stains in lymph node sections from 22 patients with non-Hodgkin's Lymphoma. Variations in the distribution and structure of glycoprotein sequences between the different types of lymphoma, and also normal nodes, have been detected.**

**The lectin-binding patterns of neoplastic lymphocytes of small cell lymphomas, both follicular and diffuse, suggested a predominance of sialylated glycopeptides, as in normal small lymphocytes of the mantle zone of germinal centres. In contrast, the staining patterns of large cell follicular and diffuse lymphomas showed a greater diversity of carbohydrate structure, with enhanced cytoplasmic staining and increased numbers of incomplete oligosaccharide sequences. Heterogeneity of staining, together with reduced sialic acid expression at all cellular sites was a common feature of lymphoblastic lymphomas and seemed to be linked with a poor prognosis.**

**The extracellular matrix of small cell follicular lymphomas showed altered saccharide content, but retained some degree of organization. The large cell follicular lymphomas were characterized by a prominent disorderly matrix, with staining characteristics which suggested shedding of surface membrane from component cells. The loss or disordered production of the normal extracellular matrix may reflect a breakdown of control mechanisms within neoplastic follicles.**

A TAXONOMY of lymphomas should contribute to an understanding of the basic biology and pathogenesis of the disease. Ideally it should be able to separate these neoplasms from non-malignant conditions and predict their clinical behaviour, response to treatment and ultimate prognosis. In recent years, 6 major classifications of NHDL (Rappaport, 1966; Lennert & Mohri, 1978; Lukes & Collins, 1977; Bennett *et al.*, 1974; Dorfman, 1974; Mathé *et al.*, 1976) have been proposed and all seem to have prognostic relevance (Berard *et al.*, 1980). Some authors (Lennert & Mohri, 1978; Lukes & Collins, 1977) have attempted to relate their classifications of these neoplasms to the immunology and function of the lymphoreticular system, and provide a better understanding of the basic biology of the disease. However, the paucity of distinctive cytological features displayed by the lymphocyte imposes limitations on standard morphology, even if this is supplemented by ultrastructural studies.

Surface marker studies have made a considerable contribution to our understanding of the pathogenesis of malignant lymphomas, and in some instances have identified the immunologic and cytologic counterpart of the malignant lymphoid cell in normal nodes (Berard *et al.*, 1980; Habeshaw *et al.*, 1979; Stein *et al.*, 1979; Lukes & Collins, 1977). The clonal origin of the majority of B cell lymphomas has been confirmed (Levy *et al.*, 1977; Mann *et al.*,

1979). However, surface marker studies have many limitations and the technical problems associated with their determination have been reviewed (Seligmann *et al.*, 1977; Lukes *et al.*, 1978). Despite their specificity monoclonal antibodies share many of these drawbacks. An alternative approach to exploring cell surface properties is the use of lectins which also display high specificity in their interactions with surface molecules, but bind to oligosaccharide sequences rather than the peptide moiety of surface glycoproteins, and may therefore give information complementary to surface marker studies. Alterations in the microenvironment of lymph nodes may be as important to the neoplastic process as changes in the surface chemistry of lymphoma cells, and it is possible to explore cell-matrix interactions by the use of lectins as histochemical stains (Bramwell *et al.*, 1982). This technique also gives information about the distribution of glycoconjugates in subcellular structures such as the nuclear membrane, nucleolus and cytoplasmic organelles.

The aim of this study was to compare the lectin staining properties, within an individual case or homogeneous groups of lymphomas, observed with a panel of lectins covering the common constituent sugars. These patterns of staining give some indication of the distribution, structure and sequence of oligosaccharides, which may have relevance to the neoplastic process.

### MATERIALS AND METHODS

*Lymphoid tissues.*—Lymph nodes were obtained from 22 patients who underwent biopsy for diagnostic or staging purposes and processed as previously described (Bramwell *et al.*, 1982).

A histological diagnosis was made on formalin and methanol fixed sections using conventional stains—haematoxylin and eosin, periodate–Schiff/alcian blue, reticulin, methyl green pyronin (Bancroft & Stevens, 1977).

*Lectin staining procedure.*—The lectins used and details of the technique are described in the previous paper (Bramwell *et al.*, 1982).

### RESULTS

This work has been described in more detail (Bramwell, 1981).

### (1) *Follicular lymphomas*

A follicular pattern was evident in 9 specimens, and the histological subclassification according to Rappaport & Kiel is shown in Table I.

TABLE I.—*Histological classification—follicular lymphomas*

| Patient | Rappaport | Kiel |
|---|---|---|
| C.H. | NPDL | CB/CC/Sc |
| W.C. | NPDL | CB/CC/Sc |
| F.B. | NPDL (+ diffuse areas) "signet ring" variety | CB/CC/Sc (+ diffuse areas) "signet ring" variety |
| E.O. | NM (+diffuse areas) | CB/CC/Lc (+ diffuse areas) |
| M.N. | NM | CB/CC/Lc |
| R.S. | NM | CB/CC/Lc |
| P.K. | NM | CB/CC/Lc |
| A.S. | NH | CB/CC/Lc |
| E.V. | NH | CB/CC/Lc |

Abbreviations
NPDL = Nodular poorly differentiated lymphocytic
NM = Nodular mixed
NH = Nodular histiocytic
CB/CC/Lc = Centroblastic/centrocytic large cell, follicular
CB/CC/Sc = Centroblastic/centrocytic small cell, follicular

The results of lectin staining are summarized in Table III and illustrated in Fig. 1.

(*a*) *Centroblastic/centrocytic small cell* (C.H., W.C., F.B.).—In all 3 cases the majority of cells, both within and outwith follicles, were small and showed weak surface staining by F-Con A, F-LCA, F-RCA, F-WGA, F-LTA, F-PNA, F-SBA and F-DBA, although scattered larger cells displayed brighter cytoplasmic staining by the first 3 lectins. In contrast, this predominant population of small cells showed bright staining of the nuclear membrane and chromatin by F-LA, F-PWM and F-PHA, but interspersed there were larger, weaker cells, particularly in follicles. In C.H. and W.C., an orderly, fine filamentous extracellular matrix within follicles was stained only by

FIG. 1.—Follicular lymphoma, × 700. F-RCA. (A) Centroblastic/centrocytic small cell; (B) centroblastic/ centrocytic large cell.

F-RCA and F-WGA. F.B. differed from the other 2 nodes in having a prominent disorderly matrix, which, with the exception of F-PNA, F-SBA and F-DBA was well stained by all lectins. In all 3 cases, macrophages were inconspicuous.

(*b*) *Centroblastic/centrocytic large cell* (E.O., M.N., R.S., P.K., A.S., E.V.).—Compared with the small cell follicular lymphomas, M.N. and E.O. showed an increased proportion of larger cells. In all the remaining cases large cells, showing bright surface and cytoplasmic staining by F-Con A, F-LCA, F-RCA, F-PHA, and to a lesser extent F-WGA, predominated. These cells were mainly confined to follicles in R.S. and A.S., but filled follicular and interfollicular areas in P.K. and E.V. Two different patterns of staining by F-LA were visible. In R.S., P.K. and E.V. the large lymphoid cells showed reduced staining of the nuclear membrane and chromatin, but enhanced fluorescence in cytoplasm and at the cell surface. The heterogeneous cell populations visible in A.S. and E.O. contained many cells which displayed reduced fluorescence of the nuclear membrane. Staining in the cytoplasm was clearly visible but was not enhanced. Staining of the abundant, disorderly extracellular material visible within the follicles of A.S., E.O., and R.S., and in all areas of E.V. and P.K., closely resembled that of cell surface membranes

TABLE II.—*Histological classification—diffuse lymphomas*

| Patient | Rappaport | KIEL |
|---|---|---|
| I.B. | DWDL | LC |
| D.B. | DWDL | LC |
| D.E. | DWDL | LC |
| E.S. | DLI | LC |
| B.R. | Sézary syndrome | LC Sézary varient |
| L.D. | LB | LB |
| F.H. | LB | LB |
| M.B. | LB | LB |
| M.W.B. | UL | LB |
| J.D. | DH (+ nodular areas) | CB (+nodular areas) |
| M.J.C. | DH | CB |
| R.M. | DH | CB |
| J.F. | DH | IB |

Abbreviations:
DWDL = Diffuse well-differentiated lymphocytic
DLI = Diffuse lymphocytic, intermediate
LB = Lymphoblastic
UL = Undifferentiated lymphoma
DH = Diffuse histiocytic
LC = Lymphocytic
CB = Centroblastic
IB = Immunoblastic

TABLE III.—*Summary—follicular lymphomas*

| Type of lymphoma | F-CON A | F-LCA | F-RCA | F-WGA | F-LA | F-PWM | F-PHA | F-LTA | F-DBA | F-SBA | F-PNA |
|---|---|---|---|---|---|---|---|---|---|---|---|
| *Centroblastic/centrocytic small cell—2 cases*§ | | | | | | | | | | | |
| Lymphoid cells | | | | | | | | | | | |
| Follicles | 1, 2 | 1, 2 | 1, 2 | 1 | 3, 4 | 2, 3 | 3 | 2 | 1 | 1 | 1 |
| Interfollicular | 1, 2 | 1, 2 | 1, 2 | 1 | 4 | 2, 3 | 3 | 2 | 1, 2 | 1, 2 | 1, 2 |
| Extracellular matrix, little | | | | | | | | | | | |
| Follicles | 1, 2 | 1, 2 | 2, 3 | 2 | 2 | 1, 2 | 2 | 1 | 1 | 1 | 1 |
| Interfollicular | 1 | 1 | 1, 2 | 1 | 2 | 1 | 2 | 1 | 1 | 1 | 1 |
| Macrophages, small, few | 3 | 3 | 3 | 3 | 3 | 2 | 3 | 3 | 2 | 2 | 2 |
| *Centroblastic/centrocytic large cell—6 cases* | | | | | | | | | | | |
| Lymphoid cells | | | | | | | | | | | |
| Follicles | 2, 3 | 2, 3 | 2, 3 | 1, 2 | 3, 4* | 1, 2 | 2, 3† | 1, 2‡ | 1 | 1 | 1 |
| Interfollicular | 1, 2, 3 | 1, 2, 3 | 1, 2, 3 | 1, 2 | 2, 3, 4* | 1, 2, 3 | 2, 3† | 1, 2‡ | 1, 2 | 1, 2 | 1, 2 |
| Extracellular matrix, abundant disorderly | | | | | | | | | | | |
| Follicular | 3 | 2 | 2, 3 | 3 | 3, 4 | 1 | 2, 3 | 1 | 1 | 1 | 1 |
| Interfollicular | 2, 3 | 1, 2 | 2, 3 | 2, 3 | 2, 3, 4 | 1 | 2, 3 | 1 | 1 | 1 | 1 |
| Macrophages, large | 4 | 3 patchy 4 | 4 | 4 | 4 | 2 patchy 4 | 3, 4 | 3 | 2 | 2 | 3 |

* Reduced nuclear membrane, increased cytoplasmic stain.

† Reduced nuclear membrane, increased cytoplasmic stain.

‡ Increased cytoplasmic stain.

§ Third case similar, but some cells showed granular staining (F-LCA, F-PHA) and prominent disorderly matrix stained by most lectins.

***Key to intensity of fluorescence:***

Ascending scale—weak (1)→brilliant (6)

TABLE IV.—*Summary—diffuse lymphomas*

| Type of lymphoma | F-CON A | F-LCA | F-RCA | F-WGA | F-LA | F-PWM | F-PHA | F-LTA | F-DBA | F-SBA | F-PNA |
|---|---|---|---|---|---|---|---|---|---|---|---|
| *Lymphocytic—4 cases* | | | | | | | | | | | |
| Lymphocytes, small | 1, 2 | 2 | 1, 2 | 1 | 4 | 3 | 3 | 1, 2 | 1, 2 | 1, 2 | 1, 2 |
| Extracellular matrix, little | 1 | 1 | 1 | 1 | 1 | 1 | 1 | 1 | 1 | 1 | 1 |
| Macrophages, rare, small | 2, 3 | 2, 3 | 3 | 3, 4 | 3 | 2 patchy 3 | 3 | 3 | 2 | 2 | 2 |
| *Lymphoblastic (+Sézary)—4 cases* | | | | | | | | | | | |
| Lymphoblasts, heterogeneous | 1, 2, 3, 4 | 1, 2, 3 | 1, 2, 3 | 1, 2, 3 | 1, 2, 3, 4 | 1, 2, 3 | 1, 2, 3, 4 | 1, 2, 3 | 1, 2 | 1, 2 | 1, 2 |
| Extracellular matrix | | | | | | | | | | | |
| Little (1) | 1 | 1 | 1 | 1 | 1 | 1 | 1 | 1 | 1 | 1 | 1 |
| Moderate (1) | 2 | 2 | 2, 3 | 2 | 2 | 2 | 2, 3 | 1 | 1 | 1 | 1 |
| Extensive (2) | 2, 3 | 2 | 3 | 2, 3 | 2, 3 | 1, 2 | 3 | 1 | 1 | 1 | 1 |
| Hyaline (1) | 4 | 4 | 4 | 4 | 5, 6 | 4 | 5 | 4 | 3 | 3 | 3 |
| Macrophages | | | | | | | | | | | |
| Not identified (2) | | | | | | | | | | | |
| Large, vacuolated (3) | 4 | 3 patchy 4 | 4 | 4 | 4 | 3 | 4 | 3 | 2 | 2 | 3 |
| *Centroblastic/immunoblastic—4 cases* | | | | | | | | | | | |
| Centroblasts/immunoblasts | 3, 4 | 2, 3 | 3, 4 | 2, 3 | 4* | 1, 2 | 2, 3* | 2† | 1, 2 | 1, 2 | 1, 2 |
| Anaplastic centrocytes | 3 | 2 | 3 | 2 | 3, 4* | 1, 2 | 2, 3* | 2† | 1, 2 | 1, 2 | 1, 2 |
| Small lymphocytes (present 3) | 1, 2 | 1, 2 | 1, 2 | 1 | 4 | 2, 3 | 3 | 2 | 2 | 2 | 2 |
| Extracellular matrix | | | | | | | | | | | |
| Amorphous (3) | 3 | 2 | 3 | 2, 3 | 3 | 1 | 3 | 1 | 1 | 1 | 1 |
| Hyaline (1) | 4 | 4 | 4 | 4 | 6 | 4 | 5 | 4 | 3 | 3 | 3 |
| Macrophages | | | | | | | | | | | |
| Not identified (2) | | | | | | | | | | | |
| Large, vacuolated (2) | 4 | 3 patchy 4 | 4 | 4 | 4 | patchy 3 | 4 | 3 | 2 | 2 | 3 |

* Reduced nuclear membrane, increased cytoplasmic stain.

† Increased cytoplasmic stain.

FIG. 2.—Diffuse lymphoma, × 700. F-Con A. (A) Lymphocytic; (B) lymphoblastic; (C) centroblastic.

in each case. Frequent large "foamy" macrophages, present in E.O., R.S., A.S., P.K. and E.V. were marginally brighter than lymphoid cells.

(2) *Diffuse lymphomas*

Thirteen lymphomas showed a diffuse pattern, although in the 3 cases of centroblastic lymphoma (J.D., R.M., M.J.C.) previous biopsies had shown follicular areas. The histological subclassification according to Rappaport & Kiel is shown in Table II.

The results of lectin staining are summarized in Table IV and illustrated in Figs 2–4.

(*a*) *Lymphocytic* (I.B., E.S., D.B., D.E.).—The monomorphous populations of small cells in I.B., E.S. and D.B. were stained weakly by F-Con A, F-LCA, F-RCA, F-WGA, F-LTA, F-PNA, F-SBA and F-DBA and brightly by F-LA,

FIG. 3.—Diffuse lymphoma, ×700. F-LA. (A) Lymphocytic; (B) lymphoblastic; (C) centroblastic.

F-PWM and F-PHA. Extracellular material was minimal.

(*b*) *Lymphoblastic* (L.D., F.H., M.B., M.W.B.) *and Sézary* (B.R.).—All these cases were characterized by heterogeneous populations in which the fluorescence of cells ranged from weak to bright with F-Con A, F-LCA, F-RCA, F-WGA, F-LA, F-PWM and F-LTA. Although extracellular material was negligible in L.D., the remaining cases showed variable amounts of disorderly matrix which was well stained by F-Con A, F-LCA, F-RCA, F-WGA, F-LA and F-PHA.

(*c*) *Centroblastic* (J.D., R.M., M.J.C.).—All comprised homogeneous populations of large tumour cells which showed increased surface and cytoplasmic staining with F-Con A, F-LCA, F-RCA, F-WGA and F-PHA, but weak staining by F-PWM, F-PNA, F-SBA, F-DBA. Enhanced surface and cytoplasmic staining by F-LA was accompanied by a reduction in fluorescence at the nuclear membrane. In

FIG. 4.—Immunoblastic lymphoma, × 700. F-LA.

J.D. and M.J.C. abundant, amorphous extracellular material showed staining properties similar to the surface membrane. In R.M. there was extensive bright hyaline.

(*d*) *Immunoblastic* (J.F.).—As in the centroblastic lymphomas, large tumour cells with similar staining characteristics predominated, although F-LA produced weak fluorescence at all cellular sites. The lectin staining properties of cells in one area suggested that they were a residual normal population of small lymphocytes.

### DISCUSSION

The sugar specificities of the lectins used in this study have been reviewed in the previous paper (Bramwell *et al.*, 1982).

### (1) *Follicular lymphomas*

The small cell follicular lymphomas (C.H., W.C.) generally exhibited staining properties similar to small lymphocytes and centrocytes and resembled the paracortical zones of unstimulated lymph nodes (Bramwell *et al.*, 1982). As with small lymphocytes this staining pattern suggests a predominance of complete sequences of *N*-glycosidically linked complex oligosaccharides and possibly sialylated *O*-glycosidically linked sequences. In contrast the matrix within follicles seemed to contain a particularly high density of terminal galactosyl residues suggestive of a deficiency of sialic acid.

The remaining small cell follicular lymphoma (F.B.) was atypical. The prominent "extracellular matrix" which was rich in Man, terminal Gal and possibly GlcNAc may, in fact, have been caused by staining of immunoglobulin at the perimeter of vacuoles (Vernon *et al.*, 1979). The frequent granular staining by F-LCA and F-PHA in the cytoplasm of many cells might also be related to immunoglobulin production.

The tumour population within the follicles of 3 of the large cell lymphomas (P.K., R.S., E.V.) showed an increased density of Man, Gal, GlcNAc and sialic acid in cytoplasm, the surface membrane and the abundant disorderly extracellular matrix. There was less F-LA positive material in the nuclear membrane. A high membrane turnover, with increased numbers of incomplete complex oligosaccharides, and aberrant membrane flow, such that sialylated glycopeptides destined for the nucleus pass instead to the surface membrane and are shed, could account for all these findings. Most of these large cell lymphomas (E.O., A.S., P.K., R.S., E.V.) contained large macrophages which displayed staining patterns similar to the largest tumour cells. These were probably analogous to the tingeable body macrophages of the germinal centre. In contrast, the small cell lymphomas contained few, small, relative weakly stained macrophages.

Although for E.O. and A.S. the lectin staining characteristics resembled other cases in the group, the pattern of fluorescence with F-LA was rather different. The majority of tumour cells showed reduced fluorescence at the nuclear membrane, but there was no compensatory increase in surface or cytoplasmic staining, and the extracellular matrix was comparatively weak. These features suggest reduced synthesis of sialoglycopeptides. Atkinson & Bramwell (1980*a*, *b*) demonstrated reduced amounts of sialic acid at the cell surface and in cell homogenates of a variety of neoplastic cell lines, compared with their normal counterparts.

The level of fluorescence produced by PNA was very low in all methanol fixed specimens, non-neoplastic and neoplastic, and no specific localization was observed. Rose *et al.* (1980) have reported preferential binding of PNA to the germinal centres present in murine Peyer's patches. A similar pattern of binding to germinal centres and the neoplastic follicles of follicular lymphomas has been noted in human lymphoid tissue (Rose *et al.*, 1981). As these studies were carried out on frozen sections, it is possible that PNA was binding to a short-chain glycolipid, which is extracted by methanol.

(2) *Diffuse lymphomas*

There were 4 cases of lymphocytic lymphoma, and 3 (I.B., D.B., E.S.) displayed a staining pattern similar to the small cell follicular lymphomas, and it is likely that the distribution and structure of cellular glycoproteins was similar and also resembled those found in normal lymphocytes.

The centroblastic group comprised 3 cases (R.M., J.D., M.J.C.) who were biopsied at relapse. Varying degrees of nodularity had been present in the first biopsy. This group resembled the large cell follicular lymphomas, and the composition and structure of glycoconjugates in the cells and matrix was probably similar. These findings are consonant with the gradual emergence, in follicular lymphomas, of a more actively proliferating large cell component which manifests a diffuse pattern of growth (Berard *et al.*, 1978; Risdell *et al.*, 1979), and altered saccharide expression.

A striking feature of all the lymphoblastic lymphomas (L.D., F.H., M.B., M.W.B.) was the heterogeneity of the cellular staining by the lectins. This was not related to cell size, and equivalent heterogeneity was not visible in the H. & E. stained sections. Nathwani *et al.* (1976) found that lymphoblastic lymphomas of convoluted and non-convoluted types were composed of variable numbers of prolymphocytes and lymphoblasts. Prolymphocytic differentiation was more common in convoluted cell types, but could not be detected with certainty in tissue sections, although it was clearly visible on tissue imprints and bone marrow smears. A mixture of prolymphocytes and lymphoblasts, with intermediate forms could account for the heterogeneity of lectin staining, which was particularly prominent in the one convoluted T cell lymphoma (L.D.) Nathwani *et al.* (1976) found the median survival for their group of lymphoblastic lymphomas to be 8 months. All biopsies in this group of patients were taken at first presentation. L.D. died within 9 days of biopsy. Despite intensive chemotherapy, F.H. never achieved complete remission and has progressive disease 14 months from biopsy. M.B. and M.W.B. are in remission 6+ and 14+ months from biopsy, respectively.

The lectin binding patterns of 2 additional patients gave further support to the view that reduced sialylation may be correlated with a poor prognosis. The case of Sézary syndrome (B.R.) showed staining patterns that resembled those of the lymphoblastic group. In many cells there was a high density of sugars normally found in the internal regions of complete complex oligosaccharides, which coupled with reduced sialylation at all sites, may denote an aggressive tumour. Although the total duration of disease was 36 months this patient died within 6 weeks of this biopsy. Although the lectin staining patterns of the one case of immunoblastic lymphoma (J.F.) resembled those of the centroblastic group, the tumour population showed variable fluorescence with F-LA. Cell surface and cytoplasmic fluorescence were never increased and staining of the nuclear membrane was generally reduced. A combination of exposure of sugars normally found in the internal regions of complete complex oligosaccharides, together with diminished sialylation, may reflect the more aggressive potential of this tumour. This specimen was taken at presentation and the patient died of rapidly progressive lymphoma 4 months after biopsy.

### CONCLUSIONS

Although, in these studies (see also Bramwell *et al.*, 1982), the number of cases of the various histological subtypes of lymphoma is small, certain tentative conclusions may be drawn. A low content of sialyl residues, often accompanied by increased expression of saccharides normally found in the internal regions of oligosaccharide sequences, and heterogeneity of lectin staining seemed to indicate a poor prognosis.

The presence of an orderly carbohydrate rich matrix restricted to the germinal centre of lymph nodes, has not been previously demonstrated. In small cell lymphomas there are changes in saccharide content but some degree of organization remains. The matrix of large cell lymphomas shows no structural organization, and the patterns of lectin staining suggest that shed surface membrane may be a major component.

In the diffuse large cell group, centroblastic lymphomas, which in previous biopsies had shown a follicular architecture, displayed patterns of lectin staining which differed from lymphoblastic and immunoblastic lymphomas. It will be important to expand the numbers of lymphomas of all cell types, but particularly the diffuse large cell group, to determine whether the changes reported are consistent.

A relatively high level of glycosylation in the nucleolus of the Reed–Sternberg cell has been demonstrated and merits further investigation. The proportion of malignant cells in individual cases of HD varies considerably, but with increasing experience, comparison of the morphological features and lectin staining patterns may permit the identification of abnormal cells.

This work was supported by grants from the Cancer Research Campaign. We thank Dr M. Harris and Dr C. Berard for reviewing the histopathological material in this and the preceding study.

### REFERENCES

ATKINSON, M. A. L. & BRAMWELL, M. E. (1980) studies on the surface properties of hybrid cells. I. Sialyl transferase activity in homogenates of malignant and non-malignant cells. *J. Cell Sci.*, **46**, 187.

ATKINSON, M. A. L. & BRAMWELL, M. E. (1980*b*) Studies on the surface properties of hybrid cells. II. Sialyl transferase activity on the surface of malignant and non-malignant cells. *J. Cell Sci.*, **46**, 203.

BANCROFT, J. D. & STEVENS, A. (1977) Harris's haematoxylin. In *Theory and Practice of Histological Techniques.* Edinburgh: Churchill Livingston. p. 86.

BENNETT, M. H., FARRER-BROWN, G., HENRY, K. & JELLIFFE, A. M. (1974) Classification of non-Hodgkin's lymphomas. *Lancet*, ii, 405.

BERARD, C. W., COSSMAN, J. & JAFFE, E. S. (1980) Malignant lymphomas as tumours of the immune system. *Br. J. Cancer*, **42**, 1.

BERARD, C. W., JAFFE, E. S., BRAYLAN, R. C., MANN, R. B. & NANBA, K. (1978) Immunologic aspects and pathology of the malignant lymphomas. *Cancer*, **42**, 911.

BRAMWELL, V. H. C. (1981) Studies of lectin binding to normal and neoplastic lymphoid cells. *Ph.D. Thesis, University of Mancester.*

BRAMWELL, V. H. C., CROWTHER, D., GALLAGHER, J. & STODDART, R. W. (1982) Studies of lectin binding to normal and neoplastic lymph nodes. I. Normal nodes and Hodgkin's disease. *Br. J. Cancer*, **46**, 568.

DORFMAN, R. F. (1974) Classification of non-Hodgkin's lymphomas. *Lancet*, ii, 961.

HABESHAW, J. A., CATLEY, P. F., STANSFELD, A. G. & BREARLEY, R. L. (1979) Surface phenotyping, histology and the nature of non-Hodgkin's lymphoma in 157 patients. *Br. J. Cancer*, **40**, 11.

LENNERT, K. & MOHRI, N. (1978) Histopathology and diagnosis of non-Hodgkin's lymphomas. In *Malignant Lymphomas other than Hodgkin's Disease*. New York: Springer-Verlag. p. 111.

LEVY, R., WARNKE, R., DORFMAN, R. F. & HAIMOVICH, J. (1977) The monoclonality of human B-cell lymphomas. *J. Exp. Med.*, **145**, 1014.

LUKES, R. J. & COLLINS, R. D. (1977) Lukes–Collins classification and its significance. *Cancer Treat., Rep.*, **61**, 971.

LUKES, R. J., TAYLOR, C. R., PARKER, J. W., LINCOLN, T. L., PATTENGALE, P. K. & TINDLE, B. H. (1978) A morphologic and immunologic surface marker study of 299 cases of non-Hodgkin's lymphomas and related lymphomas. *Am. J. Pathol.*, **90**, 461.

MANN, R. B., JAFFE, E. S. & BERARD, C. W. (1979) Malignant lymphomas—a conceptual understanding of morphologic diversity—a review. *Am. J. Pathol.*, **94**, 105.

MATHÉ, G., RAPPAPORT, H., O'CONOR, G. T. & TORIONI, H. (1976) Histological and cytological typing of neoplastic diseases of haematopoietic and lymphoid tissues. In *WHO International Histological Classification of Tumours*, **14**. Geneva: WHO.

NATHWANI, B. N., KIM, H. & RAPPAPORT, H. (1976) Malignant lymphoma, lymphoblastic. *Cancer*, **38**, 964.

RAPPAPORT, H. (1966) *Tumours of the Haematopoietic system. Atlas of Tumour Pathology* (Sect. 3 Fasc. 8). *Armed Forces Inst. Pathol.*, **91**.

RISDELL, R., HOPPE, R. T. & WARNKE, R. (1979) Non-Hodgkin's lymphoma: A study of the evolution of the disease based upon 92 autopsied cases. *Cancer*, **44**, 529.

ROSE, M. L., BIRBECK, M. S. C., WALLIS, V. J., FORRESTER, J. A. & DAVIES, A. J. S. (1980) Peanut lectin binding properties of germinal centres of mouse lymphoid tissue. *Nature*, **284**, 364.

ROSE, M. L., HABESHAW, J. A., KENNEDY, R., SLOANE, J., WILTSHAW, E. & DAVIES, A. J. S. (1981) Binding of peanut lectin to germinal centre cells: A marker for B-cell subsets of follicular lymphoma? *Br. J. Cancer*, **44**, 68.

SELIGMANN, M., BROUET, J. C. & PREUD'HOMME, J. L. (1977) Immunologic classification of non-Hodgkin's lymphomas: Current status. *Cancer Treat. Rep.*, **61**, 1179.

STEIN, R. S., COUSAR, J., FLEXNER, J. M. & 4 others (1979) Malignant lymphomas of follicular centre cell origin in man. III. Prognostic features. *Cancer*, **44**, 2236.

VERNON, S., VOFT, R. L., NAEIM, F. & WAISMAN, J. (1979) Nodular lymphoma with intracellular immunoglobulin. *Cancer*, **44**, 1273.


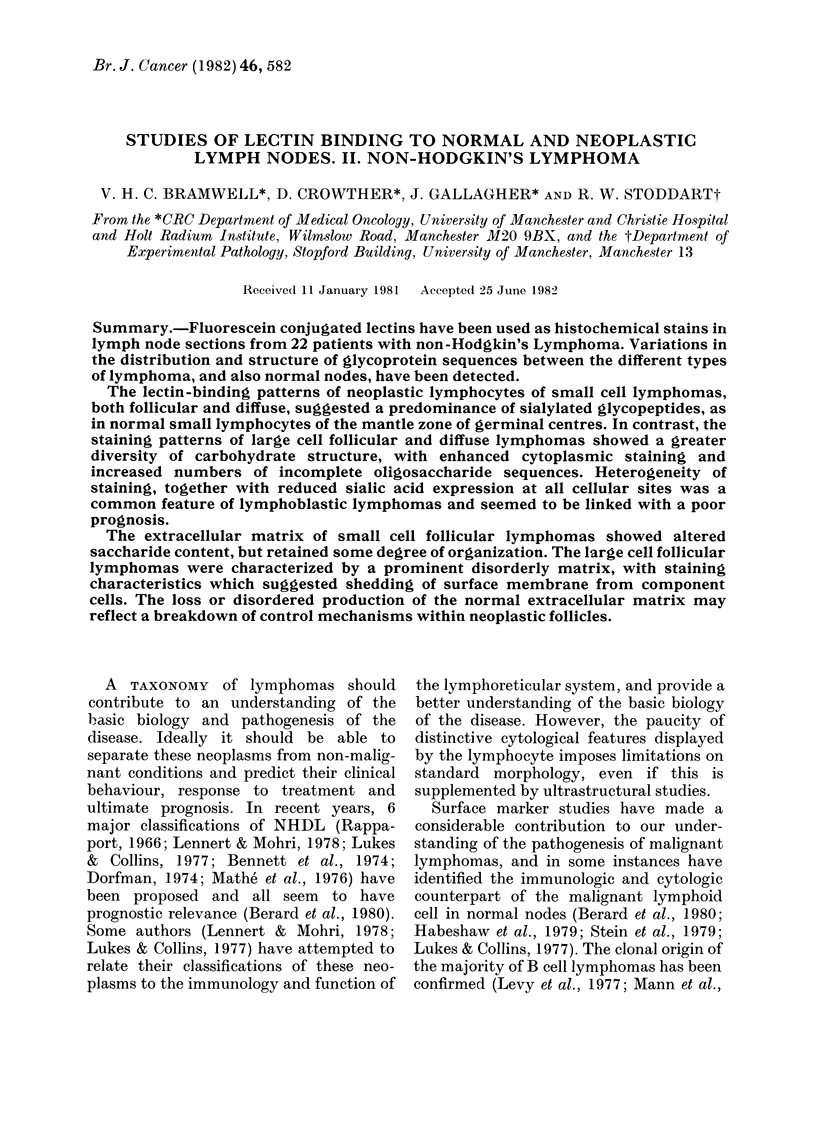

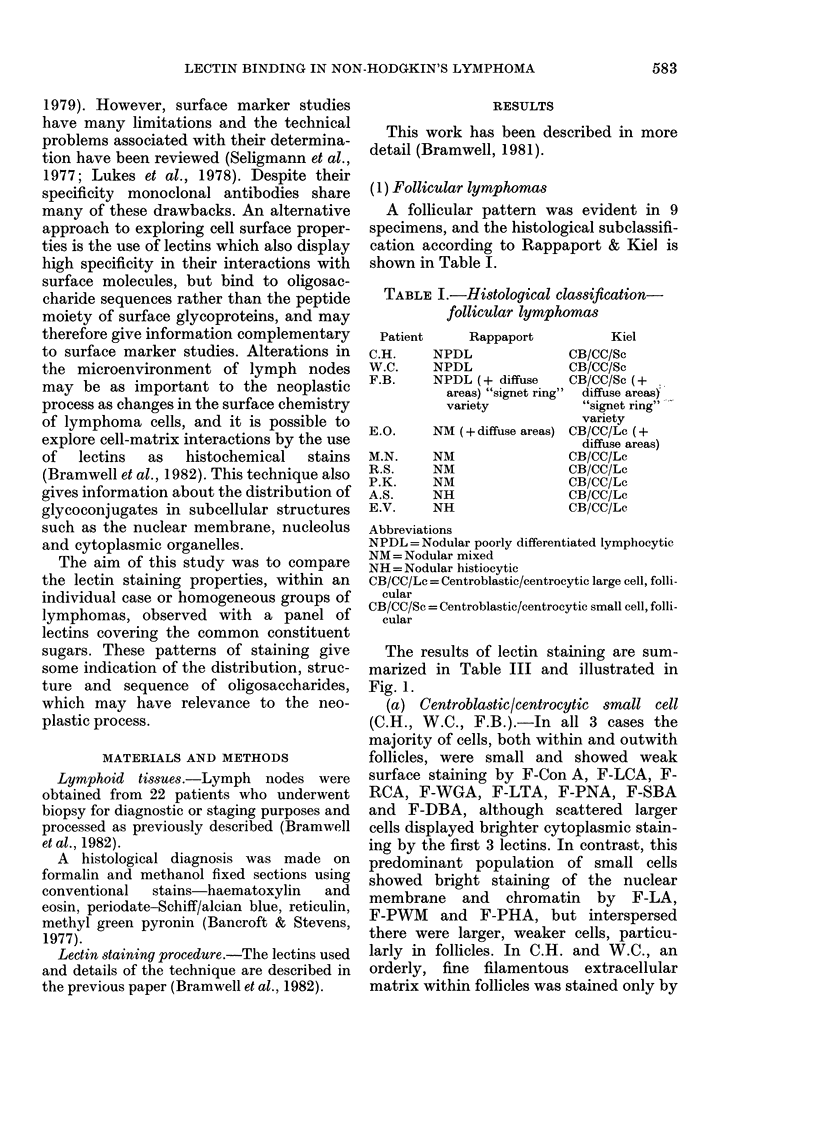

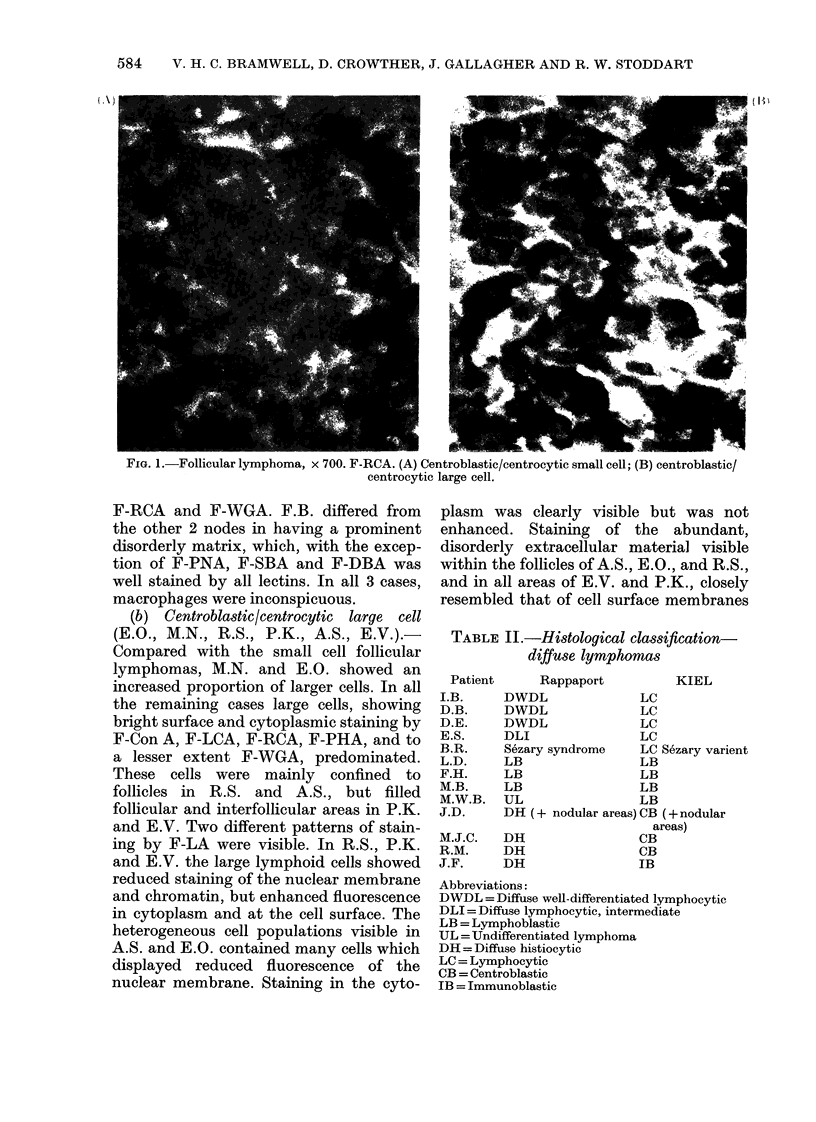

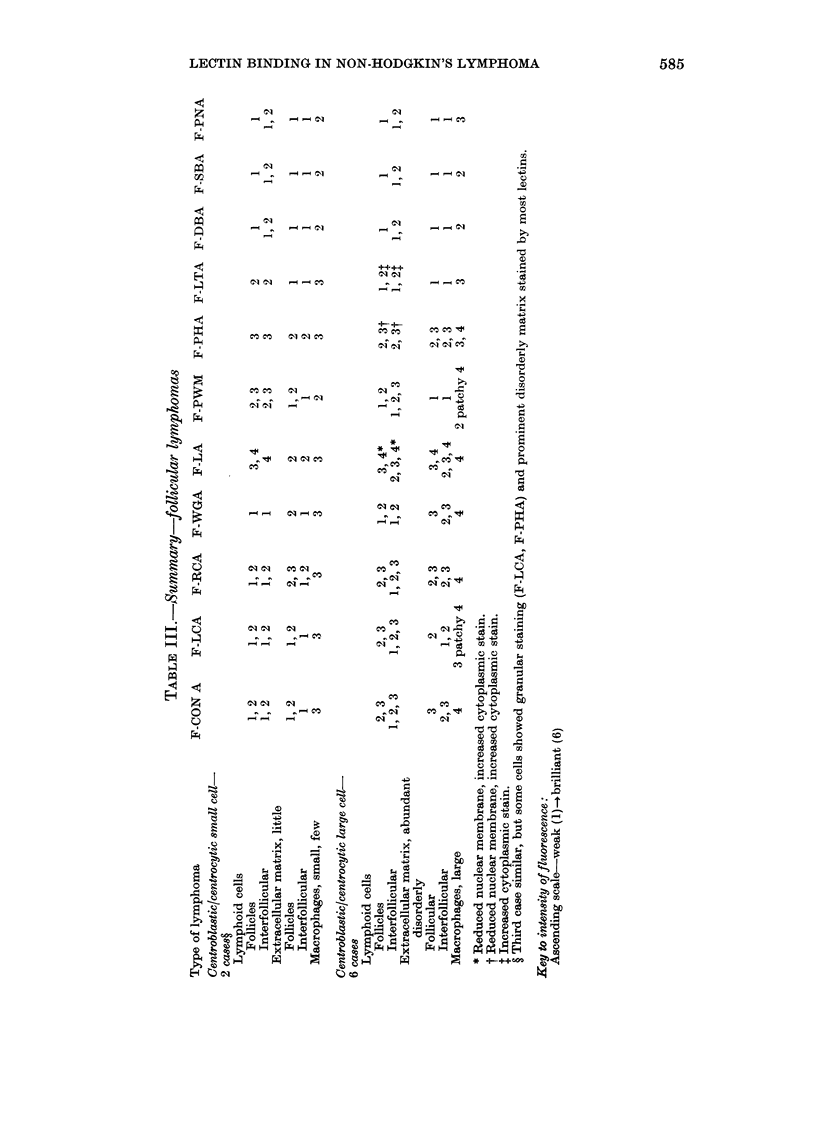

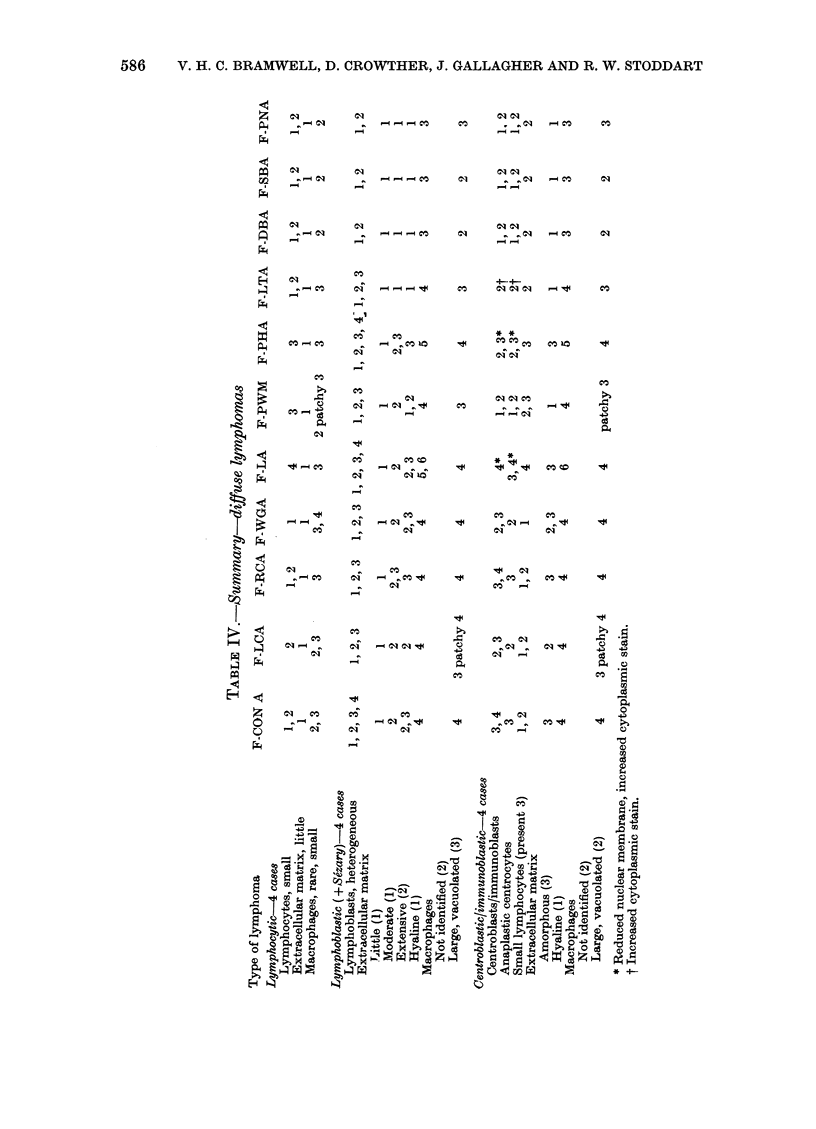

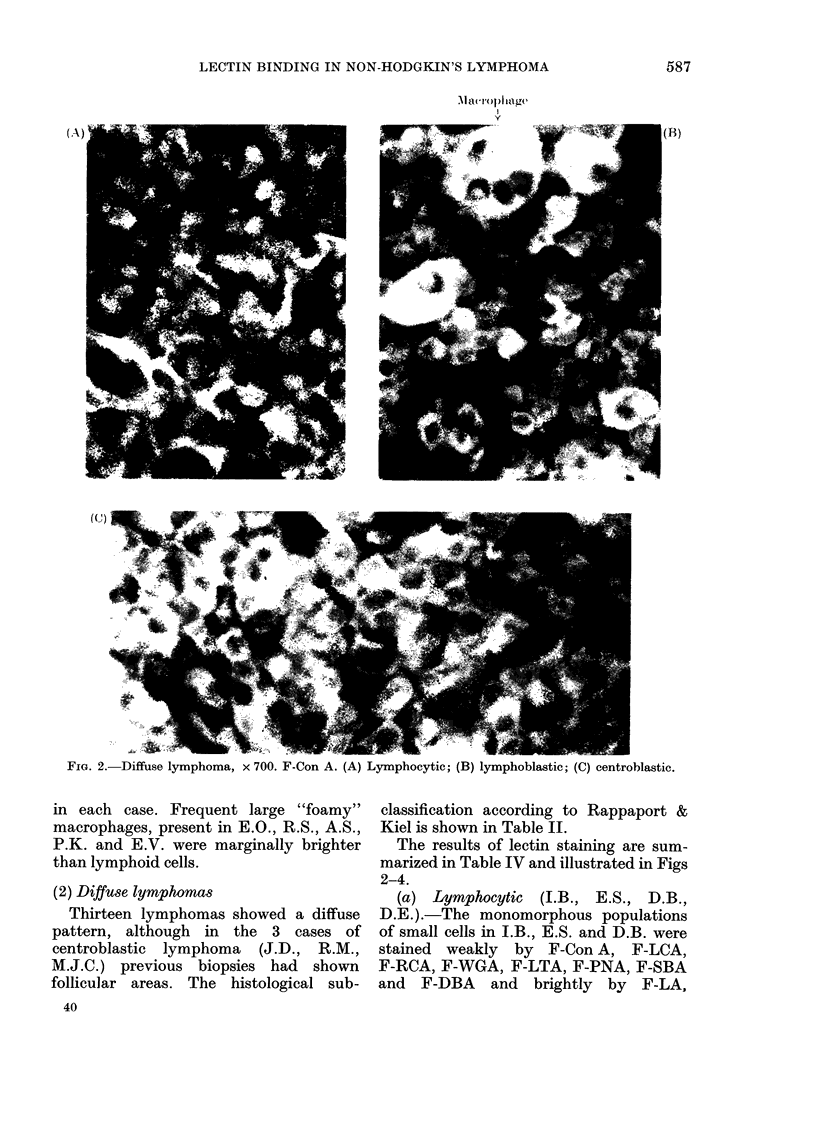

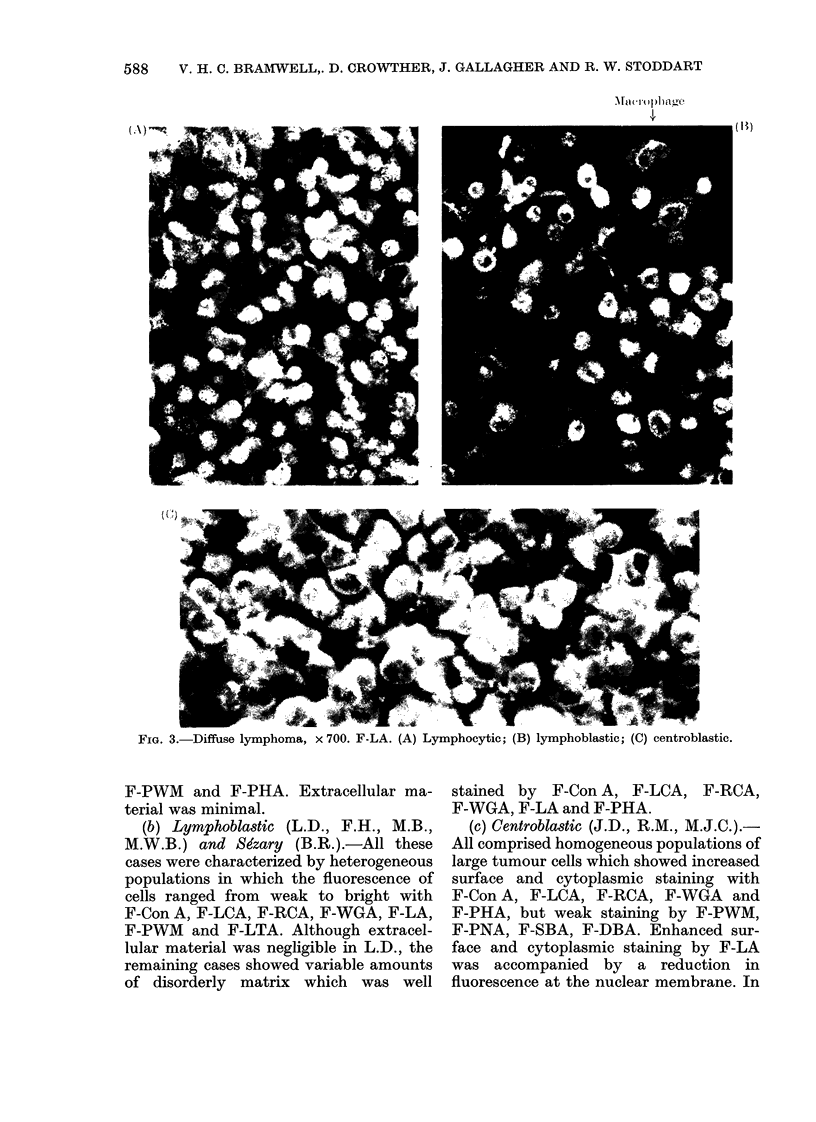

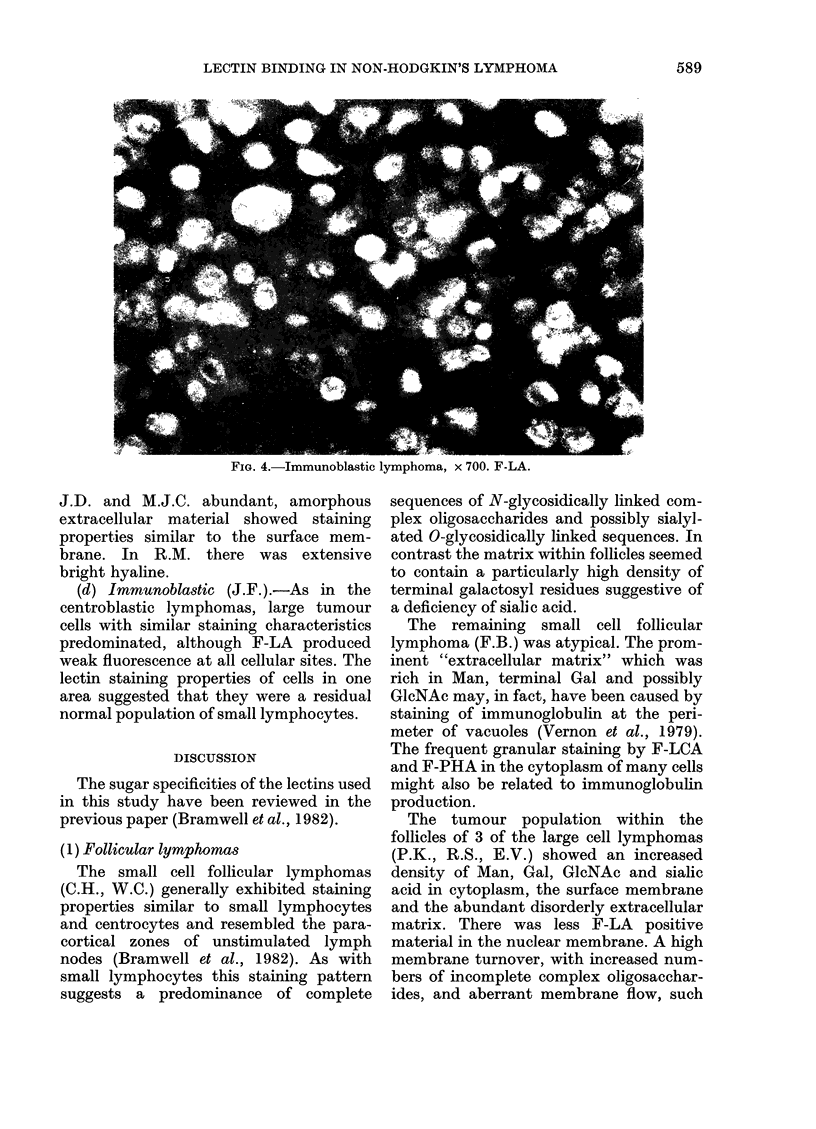

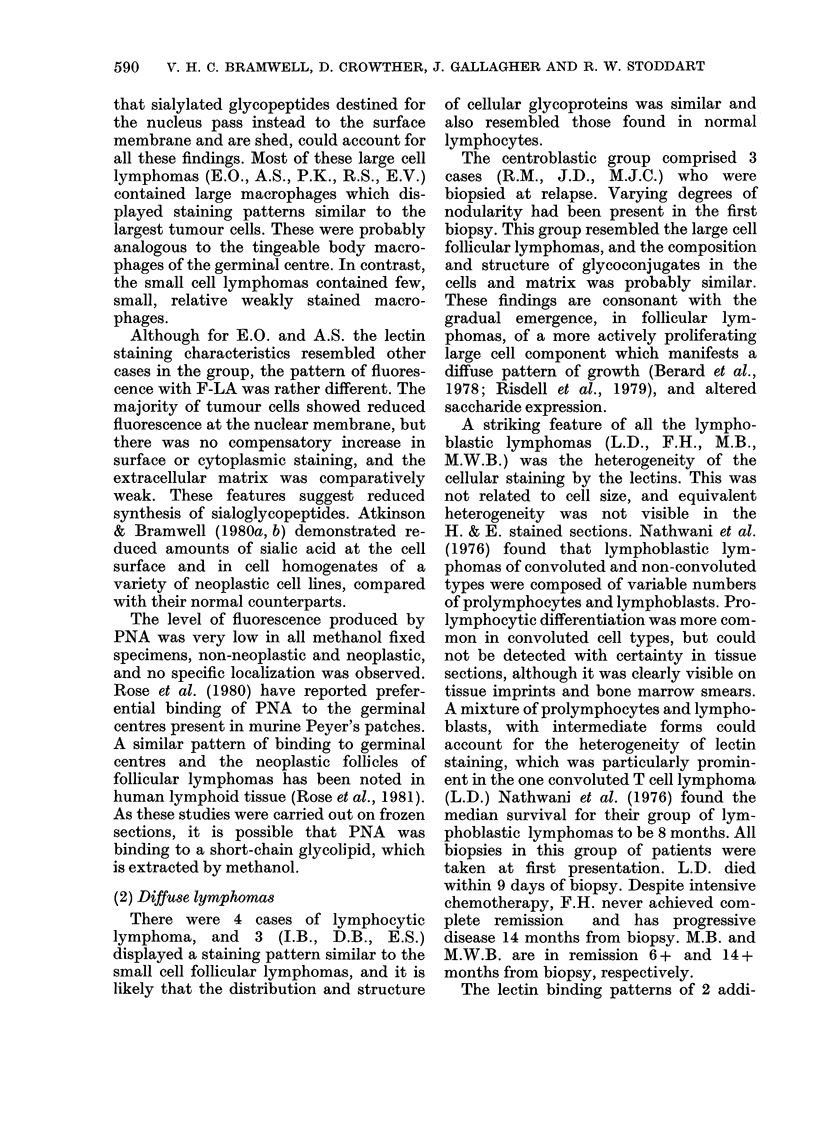

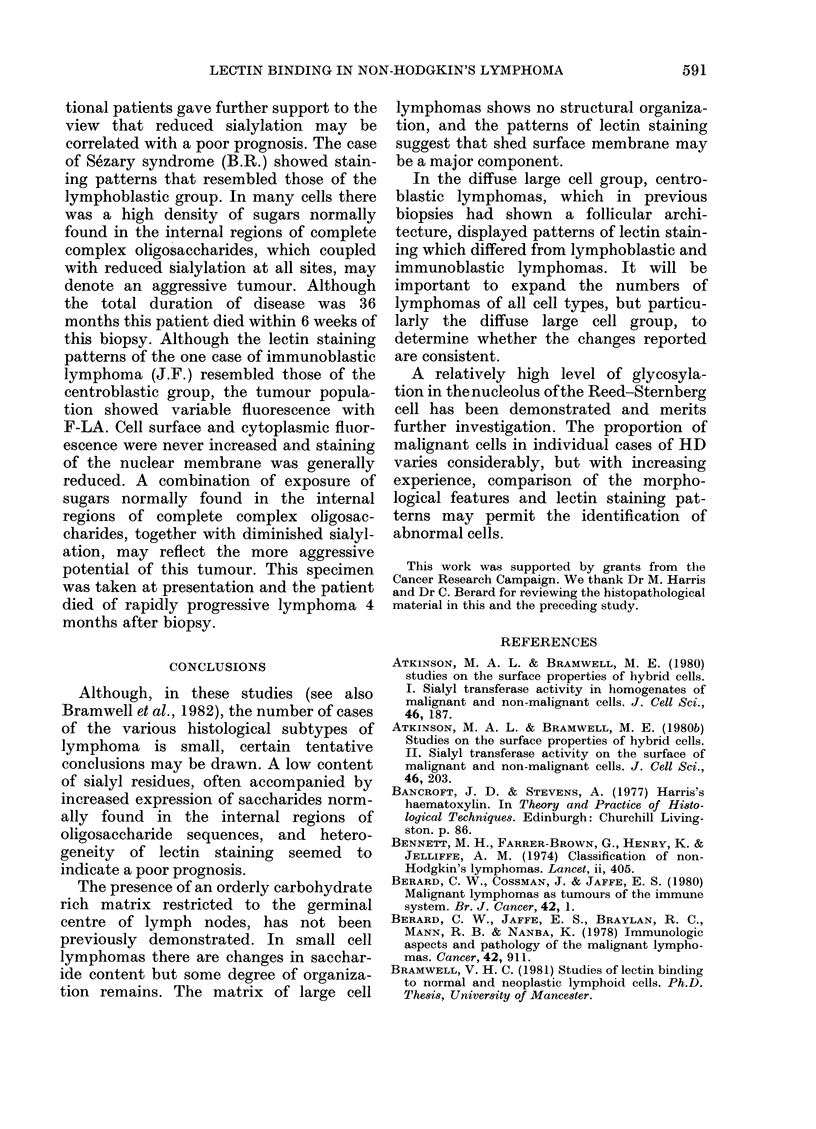

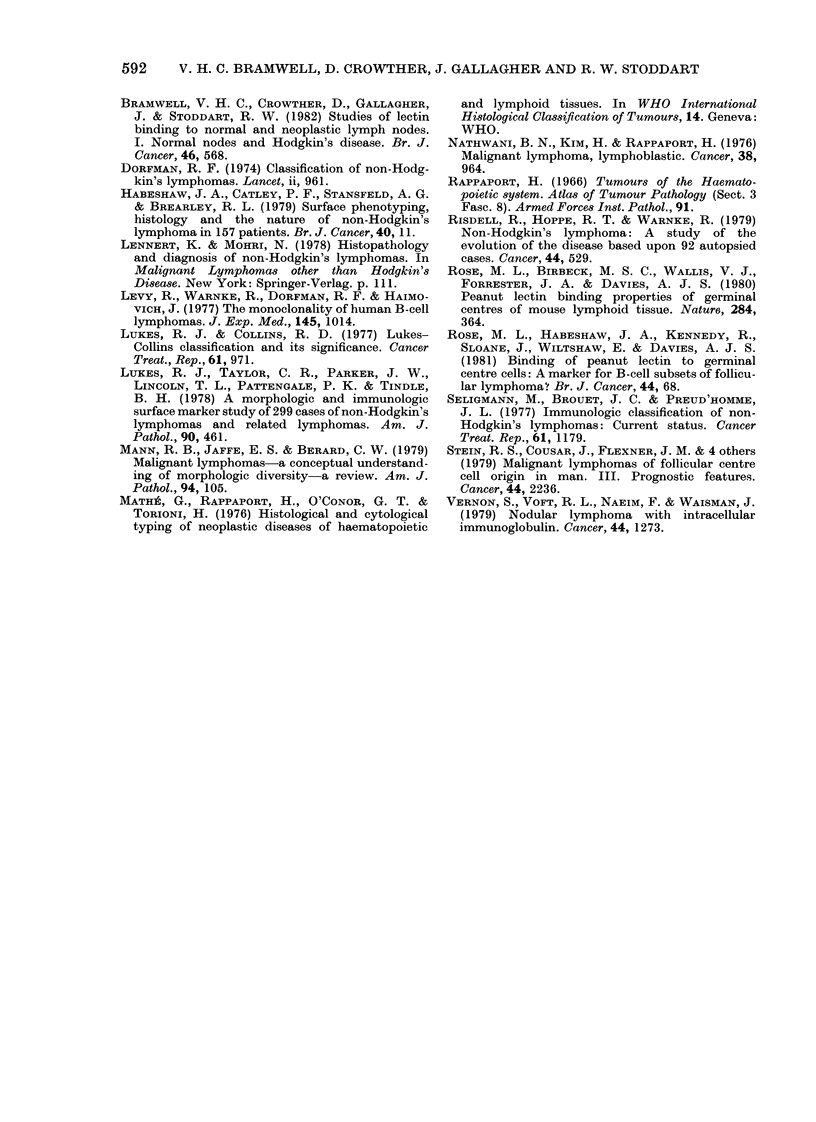


## References

[OCR_00879] Atkinson M. A., Bramwell M. E. (1980). Studies on the surface properties of hybrid cells. I. Sialyl-transferase activity in homogenates of malignant and non-malignant cells.. J Cell Sci.

[OCR_00886] Atkinson M. A., Bramwell M. E. (1980). Studies on the surface properties of hybrid cells. II. Sialyl-transferase activity on the surface of malignant and non-malignant cells.. J Cell Sci.

[OCR_00904] Berard C. W., Cossman J., Jaffe E. S. (1980). Malignant lymphomas as tumours of the immune system.. Br J Cancer.

[OCR_00909] Berard C. W., Jaffe E. S., Braylan R. C., Mann R. B., Nanba K. (1978). Immunologic aspects and pathology of the malignant lymphomas.. Cancer.

[OCR_00922] Bramwell V. H., Crowther D., Gallagher J., Stoddart R. W. (1982). Studies of lectin binding to normal and neoplastic lymphoid tissues. I. Normal nodes and Hodgkin's disease.. Br J Cancer.

[OCR_00929] Dorfman R. F. (1974). Letter: Classification of non-Hodgkin's lymphomas.. Lancet.

[OCR_00933] Habeshaw J. A., Catley P. F., Stansfeld A. G., Brearley R. L. (1979). Surface phenotyping, histology and the nature of non-Hodgkin lymphoma in 157 patients.. Br J Cancer.

[OCR_00945] Levy R., Warnke R., Dorfman R. F., Haimovich J. (1977). The monoclonality of human B-cell lymphomas.. J Exp Med.

[OCR_00950] Lukes R. J., Collins R. D. (1977). Lukes-Collins classification and its significance.. Cancer Treat Rep.

[OCR_00955] Lukes R. J., Taylor C. R., Parker J. W., Lincoln T. L., Pattengale P. K., Tindle B. H. (1978). A morphologic and immunologic surface marker study of 299 cases of non-Hodgkin lymphomas and related leukemias.. Am J Pathol.

[OCR_00964] Mann R. B., Jaffe E. S., Berard C. W. (1979). Malignant lymphomas--a conceptual understanding of morphologic diversity. A review.. Am J Pathol.

[OCR_00979] Nathwani B. N., Kim H., Rappaport H. (1976). Malignant lymphoma, lymphoblastic.. Cancer.

[OCR_00989] Risdall R., Hoppe R. T., Warnke R. (1979). Non-Hodgkin's lymphoma: a study of the evolution of the disease based upon 92 autopsied cases.. Cancer.

[OCR_00995] Rose M. L., Birbeck M. S., Wallis V. J., Forrester J. A., Davies A. J. (1980). Peanut lectin binding properties of germinal centres of mouse lymphoid tissue.. Nature.

[OCR_01002] Rose M. L., Habeshaw J. A., Kennedy R., Sloane J., Wiltshaw E., Davies A. J. (1981). Binding of peanut lectin to germinal-centre cells: a marker for B-cell subsets of follicular lymphoma?. Br J Cancer.

[OCR_01009] Seligmann M., Brouet J. C., Preud'homme J. L. (1977). Immunologic classification of non-Hodgkin's lymphomas: current status.. Cancer Treat Rep.

[OCR_01015] Stein R. S., Cousar J., Flexner J. M., Graber S. E., McKee L. C., Krantz S., Collins R. C. (1979). Malignant lymphomas of follicular center cell origin in man. III. Prognostic features.. Cancer.

[OCR_01021] Vernon S., Voet R. L., Naeim F., Waisman J. (1979). Nodular lymphoma with intracellular immunoglobulin.. Cancer.

